# Medical support during an Ironman 70.3 triathlon race

**DOI:** 10.12688/f1000research.12388.1

**Published:** 2017-08-18

**Authors:** Hae-Rang Yang, Jinwoo Jeong, Injoo Kim, Ji Eun Kim

**Affiliations:** 1 Department of Emergency Medicine , Dong-A University Hospital , 49201 Seo-Gu DaesinGongwon-Ro 26, Busan, Korea, South; 2 Department of Emergency Medicine , College of Medicine, Dong-A University 49201 Seo-Gu DaesinGongwon-Ro 26, Busan, Korea, South; 3 Department of Emergency Medical Technology, Dong-Eui Institute of Technology, 47230 Busanjin-Gu Yangi-Ro 54, Busan, Korea, South

**Keywords:** Athetic injuries, Emergency Medical Services, Sports Medicine

## Abstract

Background: The Ironman 70.3 race is also called a half Ironman, and consists of 1.9 km of swimming, 90.1 km of cycling, and 21.1 km of running. The authors provide practical insights that may be useful for medical support in future events by summarizing the process and results of on-scene medical care.

Methods: The medical post was established at the transition area between the cycling and running courses, which was close to the finish line, and staffed with the headquarters team comprised of an emergency physician, an EMT, two nurses, and an ambulance with a driver. The other five ambulances were located throughout the course. The medical staff identified participants according to their numbers when providing medical support, and described complaints, treatment provided, and disposition. When treating non-participants, gender and age were recorded instead of numbers. The treatment records were analyzed after the race.

Results: The medical team treated a total of 187 participants. One suffered cramps in the calf muscles during the swimming part of the course. Nineteen were treated for injuries suffered during the cycling race. A total of 159 were treated for injuries on the running course. Five casualties, all of which occurred during the cycling race, required transport to hospital.

Conclusions: Medical directors preparing medical support during a triathlon event should expect severe injuries in the cycling course. In hot climates, staff may also suffer from heat injuries as well as runners, and proper attention should be paid to these risks.

## Introduction

Triathlon is a sporting activity that combines swimming, cycling, and running into a single event. Triathlon events are divided into Sprint, Olympic, Long, and Ironman distances ^1^. The Ironman 70.3 race is also called a half Ironman, and consists of 1.9 km of swimming, 90.1 km of cycling, and 21.1 km of running ^2^.

While the number of mass participation sporting events is on the rise, injury data and availability of medical support plans for such events remain underreported ^3^. An understanding of the temporal and spatial characteristics of injuries sustained during triathlon races would facilitate appropriate medical support planning ^1^. However, there have been very few reports regarding medical support for triathlon events, despite the fact that the occurrence of injuries depends, to some extent, on weather conditions ^4^, and there have been no reports to date regarding medical aspects of triathlon events held in northeast Asia.

The authors were involved in planning and providing medical support for the Ironman 70.3 Busan event held in 2016. Here, we provide practical insights that may be useful for medical support in future events by summarizing the process and results of on-scene medical care.

## Methods

### Setting

The study was conducted during the Ironman 70.3 Busan race held on June 19, 2016, in the Haeundae and Gijang areas of Busan, South Korea. The Ironman 70.3 race involves 1.9 km swimming, 90.1 km cycling, and 21.1 km running. The race began at 06:45 and finished at 15:28 when the last runner crossed the finish line.

### Participants

The number of participants in the race was 765, and more than 800 staff and volunteers also took part in the event.

### Procedures

The medical support group consisted of one board-certified emergency physician as the medical director, six emergency medical technicians (EMT), four nurses, a physical therapist, three volunteers with first responder training, and six ambulances with drivers. The medical post was established at the transition area between the cycling and running courses, which was close to the finish line, and staffed with the headquarters team comprised of an emergency physician, an EMT, two nurses, and an ambulance with a driver. The other five ambulances were located throughout the course and a team with at least one EMT or nurse was allocated to each ambulance.

The emergency physician at the medical post provided on-line medical control through radio communication. Group talking using Long-Term Evolution (LTE)-based radio transceivers was the primary communication method. The medical post used another transceiver to communicate with the organizing committee. When participants required medical attention, patrols reported their location to the organizing committee and the medical post dispatched an ambulance. The medical post also provided care for those who visited the medical tent themselves.

The participants were required to report their name, gender, and age group in 5-year intervals at the time of registration, and they were assigned numbers. The medical staff identified participants according to their numbers when providing medical support, and described complaints, treatment provided, and disposition. When treating non-participants, gender and age were recorded instead of numbers. The treatment records were analyzed after the race. The temperature, humidity, and wind data measured at Haeundae weather station were downloaded from the official website of the Korean Meteorological Administration ( http://www.kma.go.kr/weather/climate/past_cal.jsp). The study was approved by the Institutional Review Board of the Dong-A University Medical Center. The need for informed consent was waived by the Institutional Review Board because of the noninvasiveness and retrospective nature of the study. All participants’ personal information was de-identified before analysis.

## Results

### Progression of the event

The swimming part of the race was reduced to 1 km because of rain and poor visibility. The race began on 06:45 with swimming, and the first participant proceeded to the cycling section at 07:04. The swimming section was closed at 08:04. The running race began at 09:25, and the entire race ended at 15:28 when the last runner crossed the finish line. The timeline of event progression and medical support activities are summarized in Table 1.

**Table 1.** Event progression and activities of the medical support teams.

Time	Event Progression	Medical Support Activities
05:30		Preparing for medical support Establishing communication
06:20		One support team (HQ) at swimming start One support team at swimming finish Four support teams move to cycling course
06:45	Swimming course start	
07:04	First player to start cycling course	One team (HQ) at swimming finish Five teams support cycling course
08:04	Last player to start cycling course Swimming course closed	HQ team moves to medical post at T2 point Five teams support cycling course
09:25	First player to start running course	HQ at T2 One team move to running course
11:15	First player at finish	Teams move to running course
12:05	Cycling course closed	HQ at T2 Five teams support running course
15:28	Last player at finish	
15:40	Event closure	

HQ: medical support headquarters. T2: Transition point between the cycling and running courses, also close to the running course finish line.

### Weather conditions

**Figure 1.**
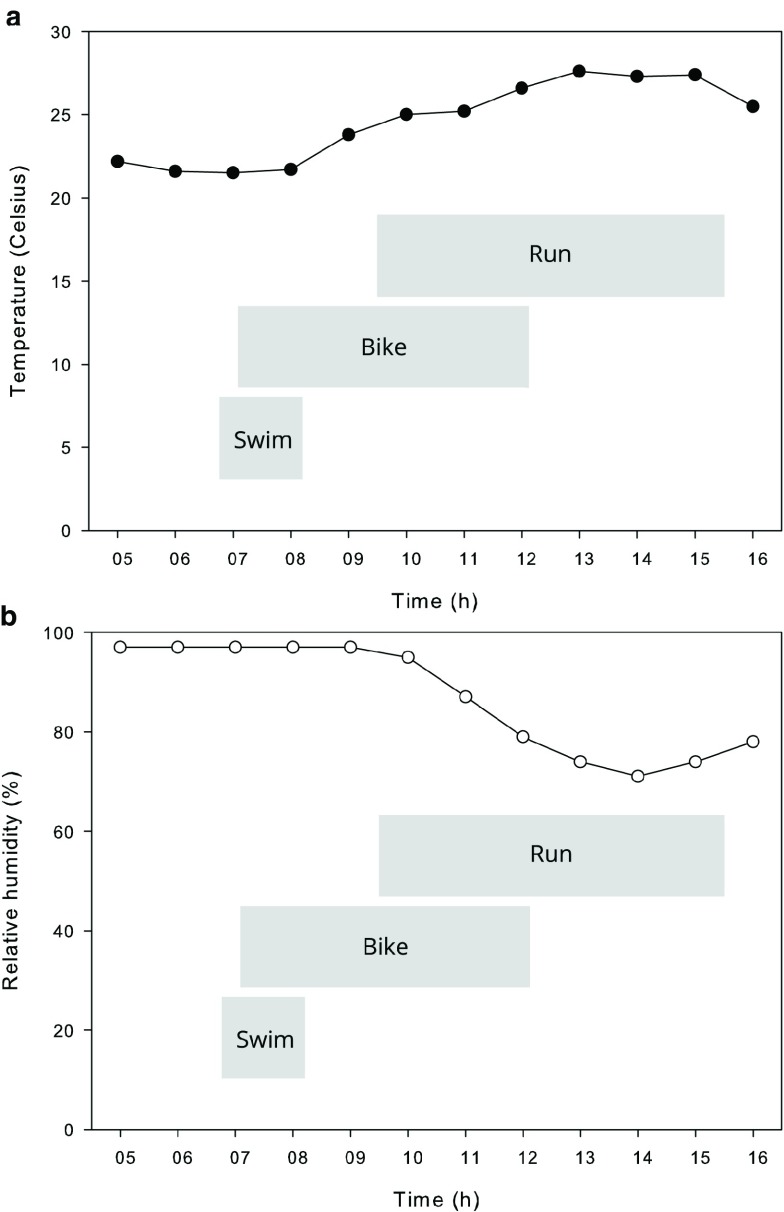
Temperature ( **a**) and relative humidity ( **b**) measured at Haeundae weather station on the day of the event.

The temperature and relative humidity data measured at Haeundae weather station are presented in Figure 1, along with progression of the event. The temperature was between 21.5°C and 27.6°C and humidity was between 71% and 97%. There was about 1 mm of precipitation around 06:00.

### Patient characteristics

The medical team treated a total of 187 participants (166 males and 21 females; dataset 1). One suffered cramps in the calf muscles during the swimming part of the course. Nineteen were treated for injuries suffered during the cycling race. A total of 159 were treated for injuries on the running course. Staff, family members, and press personnel were also treated in the medical tent. The chief complaints of the patients are summarized in Table 2.

**Table 2.** Injuries treated by the medical team during the triathlon event.

Course	Chief complaint	Number of cases
Swimming	Muscle cramps	1
Cycling	Abrasion	11
	Shoulder injury	4
	Myalgia	3
	Head injury	1
Running	Myalgia	124
	Abrasion	22
	Blister	6
	Exhaustion	3
	Laceration	2
	Sprain and strain	2
Staff	Exhaustion	3
	Headache	1
Family and press	Abrasion	2
	Foreign body	1
	Indigestion	1
	Total	187

Five casualties, all of which occurred during the cycling race, required transport to hospital. Four cases involved shoulder injuries and the other case had head trauma with brief loss of consciousness.

Participants treated by the medical support team in the Ironman 70.3 Busan race List of participants treated by the medical support team in the Ironman 70.3 Busan race held on June 19, 2016 Click here for additional data file. Copyright: © 2017 Yang HR et al. 2017 Data associated with the article are available under the terms of the Creative Commons Zero "No rights reserved" data waiver (CC0 1.0 Public domain dedication).

## Discussion

While mass participation sporting events are increasingly held in many parts of the world, there have been few reports of casualty data and medical support plans. Medical planners could utilize data from similar events as a useful guide for training and equipping their staff ^3^. Moreover, lessons learned can significantly reduce casualties in subsequent events by enabling preventive measures and improving preparedness ^4, 5^.

The pattern of injuries during a triathlon race largely depends on climate conditions, so at times cold-related injuries and at other times heat-related injuries are predominant ^4^. The weather conditions during the study period were humid and moderately hot, which were markedly different from previously reported studies in Hawaii and Australia ^1, 2, 5^, and the pattern of injuries revealed in this study would provide information facilitating the prediction of injuries in similar athletic events in Korea.

Most injuries occurring in triathlon events are minor, with blisters and abrasions as the most common types. This was also the case in the present study, considering that previous studies did not count simple myalgia among the reported injuries ^1, 5^. However, more serious injuries, such as fractures and heat-related injuries, do occur, and the organizers and medical directors should prepare for the worst case scenarios ^1, 4^.

Although swimming is considered, potentially, the most lethal part of the event, previous studies have reported lower incidences of injuries in the swimming part of these events ^1, 2^. We also found very few problems in the swimming leg of the event, which may have been partly because the swimming distance was reduced to 1 km due to the poor weather conditions. Most serious injuries occurred in the cycling portion, with five cases requiring transport to nearby hospitals ^2^. The cycling course presented challenges to the emergency responders with regard to accessing the crash sites. The injuries were notified via radio communication by the race patrols, and the exact locations were difficult to specify because of the lack of easily identifiable landmarks around the suburban public roads. Use of geographic coordinate systems with Global Positioning System (GPS) devices, such as smartphones, may improve the location of injured participants in such courses. The running part had the largest number of injury cases, as reported previously ^1^. The preceding cycling race set the stage for dehydration and exhaustion during the run, and most athletes feel that the run is the most difficult part of the race ^2^.

Three participants suffered exhaustion, and recovered with rest and oral rehydration. The incidence of heat injuries showed significant event-to-event differences, even in the same location. Such differences were reported to be caused by temperature in the preceding days, which allowed player acclimatization, in addition to preventive measures. It was reported that intravenous hydration was required for some players in the Beach2Battleship Ironman Triathlon 2014 event, in which the race length was twice that of the event included in the present study ^4^. In the 2006 Melbourne race event, three participants suffered severe heat illness and did not recover in the medical tent, so they had to be transferred to hospital. The temperature at the 2016 Melbourne event was between 21.5°C and 37.0°C, which was much higher than the temperature of 21.5°C – 27.6°C in the present study. No participants suffered heat-related collapse in the 2007 Melbourne race, with the aid of preventive measures, including an earlier start, reduced race length, increased numbers of drink stations, and increased athlete education ^5^.

In the present study, four of the staff experienced heat-related symptoms, including exhaustion and headache. In many cases of medical support for athletic events, the focus is on participating players and injuries suffered by staff have not been reported ^5, 6^. However, support staff are also exposed to a potentially hazardous environment in outdoor events, such as triathlons or marathons. The staff members have limited access to aid stations alongside the course prepared for players, because they are usually stationed at duty positions rather than moving along the course. Therefore, preventive measures, such as provision of sufficient water and education previously suggested for players ^5^, should also be carefully prepared for field staff.

In conclusion, medical directors preparing medical support during a triathlon event should expect severe injuries in the cycling course. In hot climates, staff may also suffer from heat injuries as well as runners, and proper attention should be paid to these risks.

## Data availability

The data referenced by this article are under copyright with the following copyright statement: Copyright: © 2017 Yang HR et al.

Data associated with the article are available under the terms of the Creative Commons Zero "No rights reserved" data waiver (CC0 1.0 Public domain dedication). 

Dataset 1. List of participants treated by the medical support team in the Ironman 70.3 Busan race held on June 19, 2016. Doi: 10.5256/f1000research.12388.d174190 ^7^
