# Supplementary material for: Medical support during an Ironman 70.3 triathlon race
Source: F1000Res. 2017 Aug 18;6:1516. [Version 1] doi: 10.12688/f1000research.12388.1 (PMC5583733; doi:10.12688/f1000research.12388.1)
Supplement: Peer review history

# Peer review: Referee response for version 1

**DOI:** 10.5256/f1000research.13416.r25172

**Published:** 2017-09-01

**Authors:** Gi Woon Kim

**Affiliations:** 1 Department of Emergency Medicine, Ajou University College of Medicine, Suwon, Korea, South

The article reviewed medical support activities and patient characteristics during a triathlon event. As the authors stated, injury patterns of triathlon races are very according to locations and weather conditions, and reports from eastern Asian countries such as Korea would add significant knowledge to those preparing medical support for similar events.

 However, the manuscript needs some modifications for improvement such as:

 Abstract: 

-  The acronym 'EMT' should be explained at the first appearance in the abstract.

Methods:

- The number and locations of the aid stations should be described in the Methods section, as the authors claimed in the Discussion that increased numbers of drink stations takes part in reducing heat-related injuries. 

I have read this submission. I believe that I have an appropriate level of expertise to confirm that it is of an acceptable scientific standard.

# Peer review: Referee response for version 1

**DOI:** 10.5256/f1000research.13416.r25198

**Published:** 2017-08-29

**Authors:** Woochan Jeon

**Affiliations:** 1 Department of Emergency Medicine, Inje University Ilsan Paik Hospital, Goyang, Korea, South

All contents of this article includes proper methods, analysis and conclusion.

 But, I think that if you surveyed the medical history of all participants, you could have suggested the expected risk of medical problem by a comparative analysis of two groups (health group vs medical support group).

 This article shows the importance of medical roles like number and location of medical staff in preparing sports event.

I have read this submission. I believe that I have an appropriate level of expertise to confirm that it is of an acceptable scientific standard.
